# Pathological Findings Associated With SARS-CoV-2 on Postmortem Core Biopsies: Correlation With Clinical Presentation and Disease Course

**DOI:** 10.3389/fmed.2022.874307

**Published:** 2022-07-07

**Authors:** Jose-Manuel Ramos-Rincon, Cristian Herrera-García, Sandra Silva-Ortega, Julia Portilla-Tamarit, Cristina Alenda, Francisco-Angel Jaime-Sanchez, Juan Arenas-Jiménez, Francisca-Eugenia Fornés-Riera, Alexander Scholz, Isabel Escribano, Víctor Pedrero-Castillo, Carlos Muñoz-Miguelsanz, Pedro Orts-Llinares, Ana Martí-Pastor, Antonio Amo-Lozano, Raquel García-Sevila, Isabel Ribes-Mengual, Oscar Moreno-Perez, Luis Concepcion-Aramendía, Esperanza Merino, Rosario Sánchez-Martínez, Ignacio Aranda

**Affiliations:** ^1^Internal Medicine Department, Alicante Institute for Health and Biomedical Research (ISABIAL), Alicante General University Hospital, Alicante, Spain; ^2^Clinical Medicine Department, Miguel Hernandez University of Elche, Elche, Spain; ^3^Pathology Department, Alicante Institute for Health and Biomedical Research (ISABIAL), Alicante General University Hospital, Alicante, Spain; ^4^Pathology and Surgery Department, Miguel Hernández University of Elche, Elche, Spain; ^5^Intensive Care Unit, Alicante Institute for Health and Biomedical Research (ISABIAL), Alicante General University Hospital, Alicante, Spain; ^6^Radiology Department, Alicante Institute for Health and Biomedical Research (ISABIAL), Alicante General University Hospital, Alicante, Spain; ^7^Anesthesiology Department, Alicante Institute for Health and Biomedical Research (ISABIAL), Alicante General University Hospital, Alicante, Spain; ^8^Microbiology Department, Alicante Institute for Health and Biomedical Research (ISABIAL), Alicante General University Hospital, Alicante, Spain; ^9^Pneumology Department, Alicante Institute for Health and Biomedical Research (ISABIAL), Alicante General University Hospital, Alicante, Spain; ^10^Endocrinology and Nutrition Department, Alicante Institute of Sanitary and Biomedical Research (ISABIAL), Alicante General University Hospital, Alicante, Spain; ^11^Infectious Diseases Unit, Alicante Institute for Health and Biomedical Research (ISABIAL), Alicante General University Hospital, Alicante, Spain

**Keywords:** autopsy, pathology, SARS-CoV-2, coronavirus, COVID-19

## Abstract

**Background:**

Autopsies can shed light on the pathogenesis of new and emerging diseases.

**Aim:**

To describe needle core necropsy findings of the lung, heart, and liver in decedents with COVID-19.

**Material:**

Cross-sectional study of needle core necropsies in patients who died with virologically confirmed COVID-19. Histopathological analyses were performed, and clinical data and patient course evaluated.

**Results:**

Chest core necropsies were performed in 71 decedents with a median age of 81 years (range 52–97); 47 (65.3%) were men. The median interval from symptoms onset to death was 17.5 days (range 1–84). Samples of lung (*n* = 62, 87.3%), heart (*n* = 48, 67.6%) and liver (*n* = 39, 54.9%) were obtained. Fifty-one lung samples (82.3%) were abnormal: 19 (30.6%) showed proliferative diffuse alveolar damage (DAD), 12 (19.4%) presented exudative DAD, and 10 (16.1%) exhibited proliferative plus exudative DAD. Of the 46 lung samples tested for SARS-CoV-19 by RT-PCR, 39 (84.8%) were positive. DAD was associated with premortem values of lactate dehydrogenase of 400 U/L or higher [adjusted odds ratio (AOR) 21.73; 95% confidence interval (CI) 3.22–146] and treatment with tocilizumab (AOR 6.91; 95% CI 1.14–41.7). Proliferative DAD was associated with an onset-to-death interval of over 15 days (AOR 7.85, 95% CI 1.29–47.80). Twenty-three of the 48 (47.9%) heart samples were abnormal: all showed fiber hypertrophy, while 9 (18.8%) presented fibrosis. Of the liver samples, 29/39 (74.4%) were abnormal, due to steatosis (*n* = 12, 30.8%), cholestasis (*n* = 6, 15.4%) and lobular central necrosis (*n* = 5, 12.8%).

**Conclusion:**

Proliferative DAD was the main finding on lung core needle necropsy in people who died from COVID-19; this finding was related to a longer disease course. Changes in the liver and heart were common.

## Introduction

Autopsies can shed light on the pathogenesis of new and emerging diseases. Autopsies were performed during previous coronavirus outbreaks, due to both Severe Acute Respiratory Syndrome (SARS), caused by SARS-coronavirus 1 in 2002, and Middle Eastern Respiratory Syndrome (MERS), caused by MERS-related coronavirus (MERS-CoV) in 2012. The COVID-19 pandemic, caused by SARS-coronavirus 2 (SARS-CoV-2), has resulted in more than 508 million known infections and well over 6.2 million deaths globally as of 23 April 2022 (1). COVID-19 has a high mortality rate in patients requiring hospitalization—especially older people (2, 3).

COVID-19 is a multi-organ disease that enters through the respiratory tract and especially affects the lungs, generating heterogeneous pulmonary pathologic abnormalities such as exudative diffuse alveolar damage (DAD) and organizing pneumonia (4, 5). Other organs affected by SARS-CoV-2 include the heart, liver, spleen, bone marrow, kidney, brain and testes (5–7). Different types of postmortem investigations have been performed, ranging from full autopsies to core needle necropsies (5, 7–10). The information available is generally presented in the form of smaller datasets. There are also several narrative reviews (6, 11), systematic reviews (5, 8, 11, 12) and a few meta-analyses of postmortem histopathological findings (13–15).

Conventional autopsy provides important information regarding cause of death as well as clinical and pathological correlation, and it is a paramount source of learning. Postmortem needle biopsy or core needle necropsy has an important role in diagnosis, the generation of knowledge, and quality improvement (16–18). There are few studies that analyze the clinical, analytical, and radiological factors related with postmortem findings (14).

The aim of this manuscript was to describe postmortem findings in the lung, heart, and liver tissues of decedents with COVID-19, as obtained from core needle necropsies in a single center. We also analyzed the clinical, analytical, and radiological factors related to postmortem findings, as well as virological findings (presence or not of SARS-CoV-2) in lung core needle necropsies.

## Materials and Methods

### Study Design and Setting

This cross-sectional study took place in Alicante General University Hospital (Spain) in people with COVID-19 who died from 10 March 2020 to 30 April 2021.

### Patients and Data Collection

Included patients had positive SARS-CoV-2 nasopharyngeal swabs by real-time reverse transcriptase polymerase chain reaction (RT-PCR) or antigen testing.

Patients' electronic medical records were retrospectively reviewed to collect variables including clinical characteristics, radiology imaging, and laboratory findings. We recorded demographic data, medical history, chest X-ray images, treatment received, the duration of illness, and laboratory findings (including blood count, coagulation parameters, and biochemical [C-reactive protein (CRP), lactate dehydrogenase (LDH), ferritin, d-dimer, troponin] and immunological values [interleukin-6]). Comorbidities were evaluated by means of the age-adjusted Charlson Comorbidity Index (CCI) (19). Laboratory findings were recorded on admission or diagnosis and in the 72 h prior to death. Trained physicians and radiologists collected epidemiological, clinical, and radiological data. The final X-rays before death were reviewed, and these were grouped into five categories: (1) no acute radiological findings, (2) unilateral or bilateral interstitial opacities, (3) bilateral consolidation or ground-glass like opacities, (4) consolidation with a lobar distribution, and (5) radiological findings of lung edema.

Corticosteroids, mainly dexamethasone (6 mg), were the standard of care for treating inpatients with COVID-19 pneumonia who required oxygen following the release of the results from the RECOVERY trial in July 2020. Prior to July 2020, they were used in patients with a worsening condition. Tocilizumab was used concomitantly with dexamethasone or methylprednisolone in patients with O_2_ Sat <92% (baseline or with low-flow O_2_) and C-reactive protein >7.5 mg/dL or if the patient needed high-flow O_2_, non-invasive mechanical ventilation or mechanical ventilation. Moreover, it was used in patients with a worsening condition despite treatment with dexamethasone or methylprednisolone. Treatment with remdesivir was approved by the Spanish Agency of Medicines and Medical Devices in September 2020, with common criteria for all institutions in Spain for treating patients hospitalized with COVID-19: (1) aged >12 years and >40 kg; (2) in need of supplemental low-flow oxygen; (3) ≤ 7 days from symptom onset to remdesivir prescription; and 4) meeting at least two of the following three criteria: respiratory rate ≥24 bpm, oxygen saturation at room temperature ≤ 94%, or PaO_2_ /FiO_2_ <300 mmHg. Remdesivir was administered at 200 mg on day 1 followed by remdesivir 100 mg/day on days 2–5.

#### Procurement Necropsy

With consent from the patients' families, needle core necropsies were performed on the anterior chest, obtaining two to six samples per patient within an hour of death in a negative air isolation ward with personal protective equipment and high-risk protective measures (hazard group 3) according to current protocols. Four to eight cylinders were collected for each patient with 14G core biopsy coaxial needles. The needle core necropsies performed included several organs—mainly the lungs but also the liver and heart. Procedures were performed without ultrasound guidance, but the patients' last radiographic images and surface anatomic landmarks were used as references.

#### Specimens and Pathological Examination

The tissue was fixed in neutral buffered formalin for over 24 h and then processed in line with standard biosafety measures. Two pathologists prepared hematoxylin and eosin-stained sections and examined the slides. In some cases, we performed Masson's trichrome stain and immunohistochemical stain for anti-CD4, CD8, CD20, and alfa-actin.

#### RT-PCR Assay for SARS-CoV-2 in Tissue

Samples from patients included in this study were provided by the ISABIAL BioBank, part of both the National and Valencian Biobank Networks. They were processed following standard operating procedures after approval from the cognizant ethical and scientific committees. Formalin-fixed, paraffin-embedded tissue blocks were used to prepare 20 serial sections of 4-μm thick blocks. RNA was obtained from two 10 μm paraffin-embedded tissue sections using MagCore total RNA One-Step Kit (RBCBioscience, Dublin, Ireland), an automated method that optimizes the lysis conditions to reverse the formalin fixation, without the need for overnight digestion, and that retains both large and small RNAs. The procedure was performed according to the manufacturer's instructions.

RT-PCR assays were run on the Mx3000P qPCR system with a 2019-nCoV nucleic acid detection kit (Coronavirus [COVID-19] Genesig RT-PCR assay, Primerdesign Ltd, Chandler's Ford, UK) according to the manufacturer's instructions. The target was FAM (465-510), which was simultaneously amplified and monitored during the RT-PCR assay.

Part of the research on the first 11 cases performed from March to April 2020 has been published (20).

### Statistical Analysis

Categorical and continuous variables were expressed as frequencies (percentages) and as medians (interquartile range, IQR) or means (±standard deviation), depending on the normality of the distribution. We compared pathological findings by clinical, epidemiological, and laboratory variables using the Mann-Whitney U statistic, and sex and admission to the intensive care unit (ICU) using the Chi-squared and Fisher's exact tests. Some continuous variables were dichotomized. All tests were two-sided, and *p*-values under 0.05 were considered statistically significant. The variables showing significant associations in the bivariate analysis were included in a multivariate model. Associations measured between clinical and pathological variables were presented as crude odds ratio (OR) or adjusted ORs (AOR), along with 95% confidence intervals (CI). IBM SPSS Statistics v25 (Armonk, NY, USA) was used for analyses.

### Ethics Approval

Patients' families gave their approval to perform needle core necropsies on the chest. The Ethics Committee of the Alicante General University Hospital (Spain) approved the project (PI2020-067). The study was conducted in accordance with the Declaration of Helsinki (2013), the standards of Good Clinical Practice and current legislation in Spain regarding this type of study. Data collection was carried out in accordance with the provisions of the Organic Law 3/2018 of 5 December 2018 on the Protection of Personal Data and guarantee of digital rights and Regulation (EU) 2016/679 of the European Parliament.

## Results

### Patient Characteristics

Of the 2,188 patients admitted for COVID-19 during the study period, 288 died (case fatality rate 11.2%). Chest needle core necropsies were performed in 71 (24.7%) of the deceased patients. Patients' median age was 81 years (range 52 to 97), and 47 (65.3%) were men. The median interval from symptoms onset to death was 17.5 days (range 1 to 84), and 21 (29.6%) died in the ICU. Table 1 shows the main epidemiological characteristics of the deceased patients. Supplementary Table S1 contains further details on patient characteristics, and Supplementary Table S2 presents the results of the laboratory analyses at admission and before death.

**Table 1:** Epidemiological and clinical characteristics of patients who died with COVID-19, March 2020 to April 2021.

**Variables**	**Patients (*N* = 71)**
**Demographic variables**	
Age in years, median [IQR]	81 [69, 87]
Aged >80 years, *n* (%)	36 (50.7)
Men, *n* (%)	48 (67.6)
Race/ethnicity	
White, *n* (%)	65 (91.5)
Latin American, *n* (%)	5 (7.0)
North African, *n* (%)	1 (1.4)
Institutionalized in residence, *n* (%)	7 (9.9)
Nosocomial COVID-19, *n* (%)	11 (15.5)
**Comorbidities**	
Body mass index^a^, kg/m^2^, mean ± SD	28.82 ± 4.43
Overweight (BMI> 25 kg/m^2^), *n* (%)	47 (66.2)
Obesity (BMI> 30 kg/m^2^), *n* (%)	20 (28.2)
Smoker or ex-smoker^b^, *n* (%)	24 (34.7)
> 40 pack-years, *n* (%)	9 (12.7)
Arterial hypertension, *n* (%)	51 (71.8)
Diabetes mellitus, *n* (%)	31 (43.7)
Pulmonary disease, *n* (%)	24 (33.8)
Cardiovascular disease, *n* (%)	28 (39.4)
Age-adjusted Charlson comorbidity index > 3, *n* (%)	57 (80.3)
Clinical Frailty Scale^c^, Frail (≥4), *n* (%)	47 (67.1)
**Administrative variables**	
Interval from symptoms onset to admission, days, median [IQR]	5 (2, 7)
Interval from symptoms onset to death, days, median [IQR]	17 (8, 27)
Onset-to-death interval > 15 days, *n* (%)	39 (54.9)
Length of hospital stay, days, median [IQR]	11 (5, 20)
Admitted to ICU, *n* (%)	21 (29.6)
Need for orotracheal intubation, *n* (%)	20 (28.2)
**Clinical presentation on admission**, ***n*** **(%)**	
Dyspnea	51 (71.8)
Dry cough	35 (49.3)
Fever	32 (45.1)
Asthenia	22 (31.0)
Productive cough	12 (16.9)
**Vital signs on admission**, ***n*** **(%)**	
Temperature >38°C	12 (16.9)
Oxygen saturation <90%	27 (40.9)
Tachypnea (>20 bpm)	29 (60.4)
Hypotension (systolic blood pressure <100 mmHg)	6 (8.6)
Tachycardia (>100 bpm)	24 (33.8)
PaO2 /FiO2 <300	34 (50.7)
Pre-mortem chest X-ray findings^*^, ***n*** **(%)**	
No acute pathological findings	8 (11.3)
Unilateral or bilateral interstitial infiltrates	36 (50.7)
Bilateral pneumonia	25 (35.2)
Lobar pneumonia	4 (5.6)
Acute lung edema	6 (8.5)
**Treatment**, ***n*** **(%)**	
Non-invasive mechanical ventilation^†^	44 (62.0)
High-flow nasal cannula oxygen	34 (47.9)
Continuous positive airway pressure	11 (15.5)
Bilevel positive airway pressure	1 (1.4)
Antibiotic therapy (> 48 h)	63 (88.7)
Corticosteroids	62 (87.3)
Tocilizumab	30 (42.3)
Remdesivir	5 (7.0)
Convalescent plasma	5 (7.0)
**Cause of death**, ***n*** **(%)**	
Secondary to COVID-19	63 (88.7)
Cardiovascular	2 (2.8)
Other infection	4 (5.6)
Others	2 (2.8)

*IQR, interquartile range; SD, standard deviation; ICU, intensive care unit; PaO2, arterial oxygen partial pressure in mmHg; FiO2, fractional inspired oxygen*.

* *Some patients had more than one radiological pattern*.

† *Some patients received more than one type of mechanical ventilation at different times over the course of their disease. Missing:*

a *16*,

b *1*,

c *1*.

### Histopathological Findings

**Figure 1:**
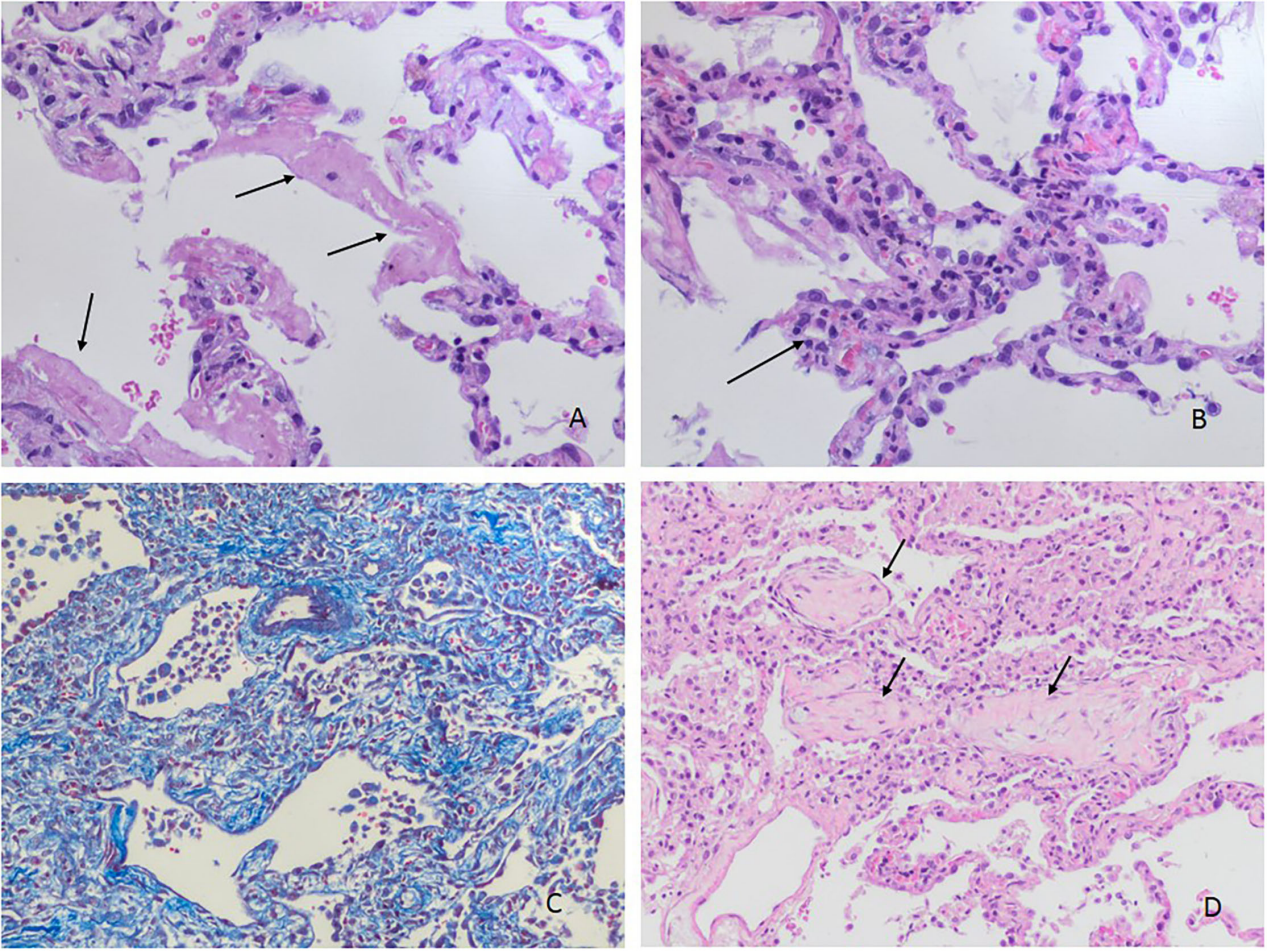
Histopathological changes in the lungs. **(A)** Hyaline membranes (arrows) without evident inflammatory infiltration (H&Ex200). **(B)** Acute inflammation in alveolar septa (capillaritis) (arrow) and type 2 pneumocyte hyperplasia without fibroblastic proliferation (H&Ex200). **(C)** Proliferative phase of diffuse alveolar damage (Masson's trichrome staining) (H&Ex100). **(D)** Polypoid plugs of fibroblastic tissue in organizing pneumonia (arrows) (H&Ex100).

**Figure 2:**
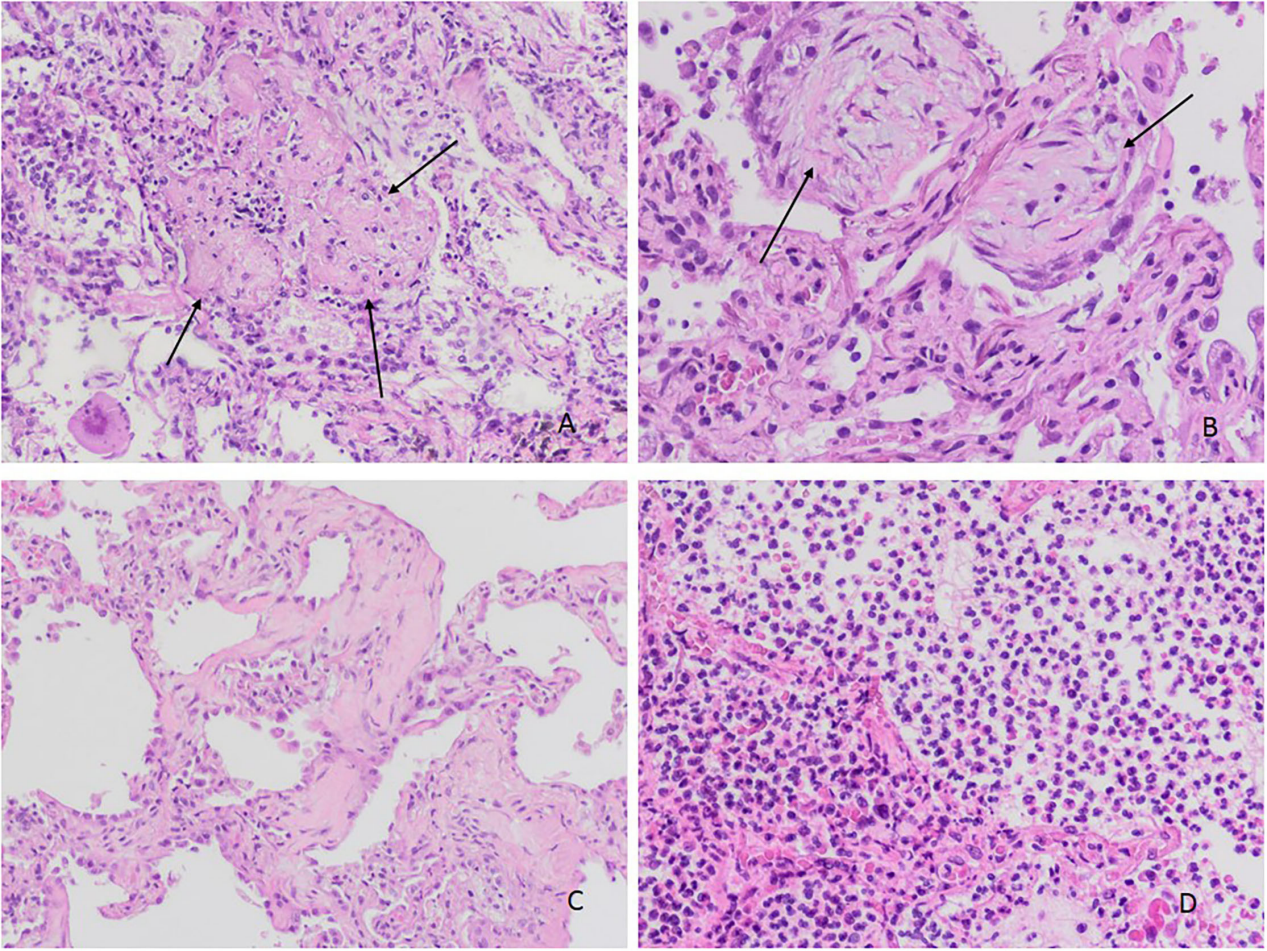
Histopathological changes in the lungs. **(A)** Acute fibrinous organizing pneumonia (AFOP): deposits in the form of “fibrin balls” in alveolar ducts and alveoli (arrows) (H&Ex100). **(B)** Organizing pneumonia. Airspaces and interstitium with fibroblastic tissue (arrows) (H&Ex200). **(C)** Fibrosing pattern with interstitial thickening, fibrosis and collagen deposit (H&Ex200). **(D)** Changes in bronchopneumonia with prominent neutrophilic infiltration in alveolar spaces (H&Ex100).

**Figure 3:**
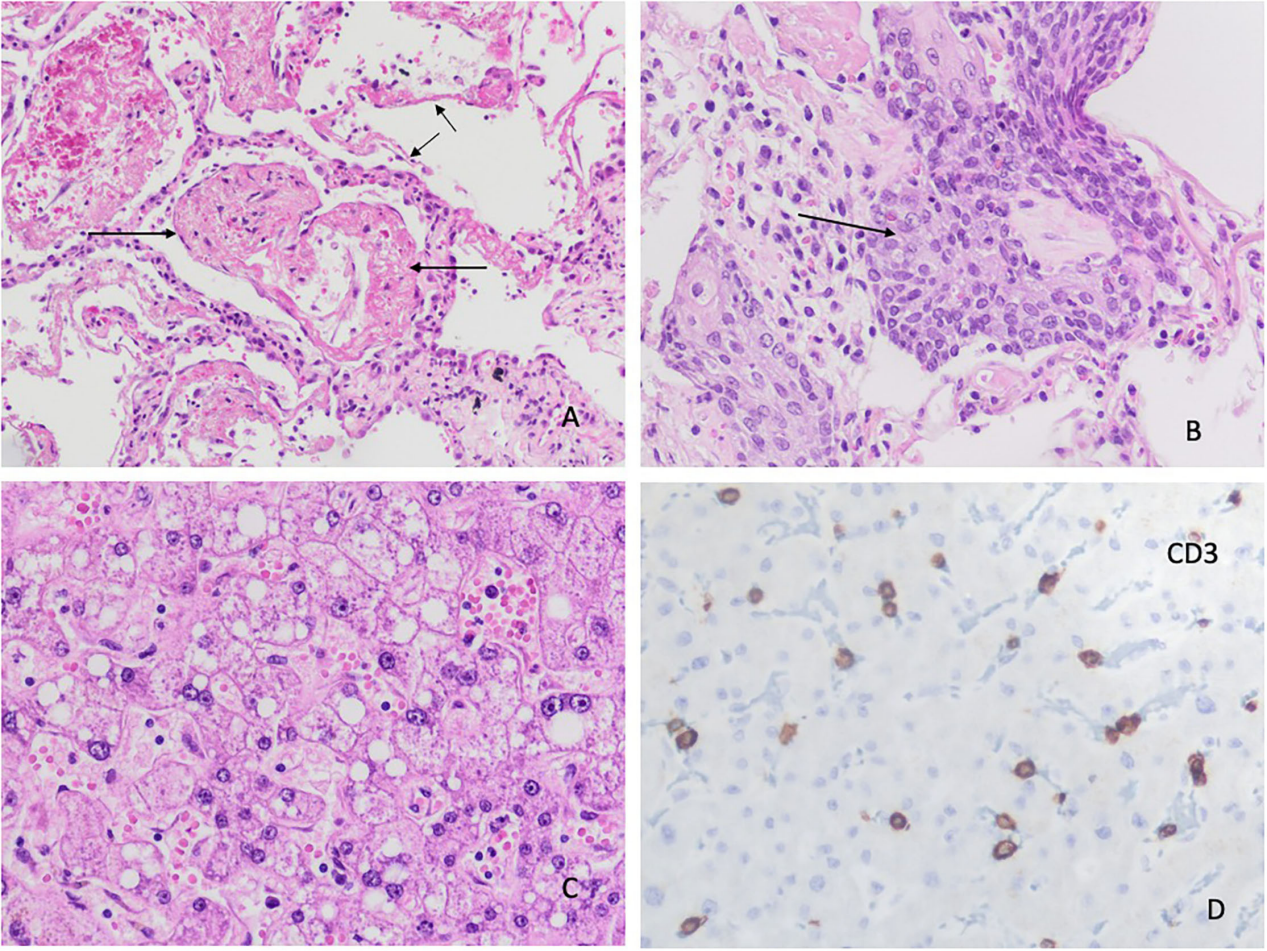
Histopathological changes in the lungs. **(A)** Lung parenchyma with interstitial mononuclear infiltrates and pneumocyte hyperplasia. No signs of thrombosis or vasculitis are observed in the vessels (arrows) (H&E x 200). **(B)** Interstitial infiltrate with predominance of CD3 (+) T lymphocytes (IHC x 100). **(C)** Predominance of CD8 (+) T lymphocytes (IHC x200). **(D)** Lesser number of CD4 (+) T lymphocytes (IHC stain x200).

**Figure 4:**
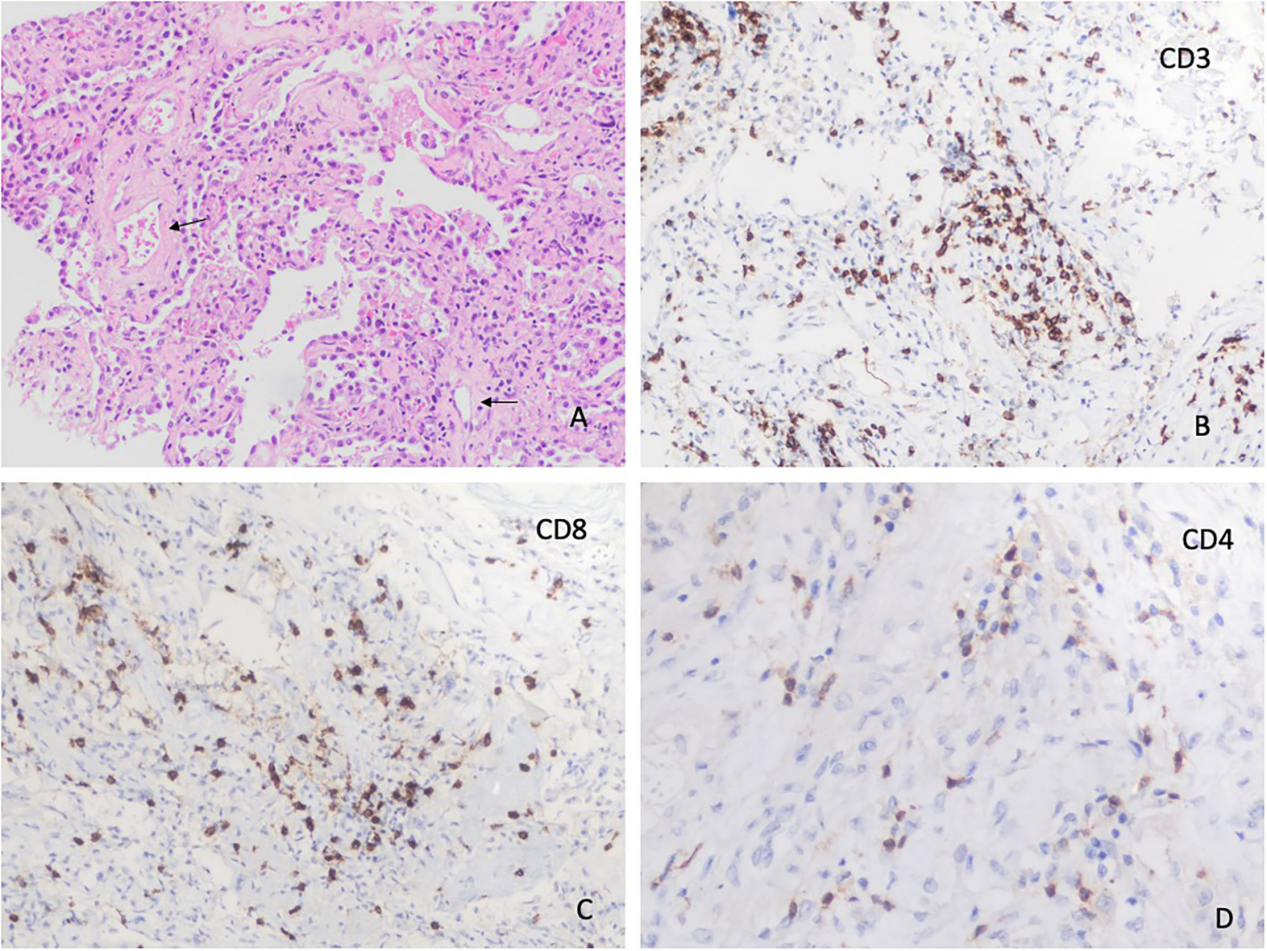
Histopathological changes in the lungs. **(A)** Lung parenchyma showing hyaline membranes (short arrows) coexisting with Acute Fibrinous Organizing Pneumonia (AFOP) (H&Ex100). **(B)** Difuse alveolar damage with squamous metaplasia (H&Ex200). Histopathological changes in the liver. **(C)** Hepatic microvesicular steatosis and lobar lymphocytic infiltrates (H&Ex200). **(D)** Hepatic lobular infiltrates with CD3 (+) T lymphocytes (IHC stain x400).

We obtained 62 (87.3%) lung samples, 48 (68.1%) heart samples, and 39 (54.9%) liver samples. Of the 62 lung samples (Figures 1–4), 51 (82.3%) were abnormal, usually due to interstitial infiltrate (*n* = 50, 80.6%) (Figure 3A), mostly by lymphocytes (Figures 3B–D). Other abnormal findings were diffuse pneumocyte hyperplasia (Figures 1A,B, 3A) (*n* = 40, 64.5%), interstitial fibrosis (*n* = 38, 61.3%) (Figures 2B,C) and alveolar fibrosis (*n* = 31, 50.0%) (Figure 2A, Table 2). The main histopathological finding was diffuse alveolar damage (DAD) (*n* = 41; 66.1%), including proliferative (*n* = 19, 30.6%; Figure 1C), exudative (*n* = 12, 19.4%), or mixed (*n* = 10, 16.1%) forms. There were 12 (19.4%) cases of acute pneumonia (Figure 2D), 10 (16.1%) of acute fibrinous and organizing pneumonia (AFOP) (Figure 1D); and 3 (4.8%) organizing pneumonias (Table 2).

**Table 2:** Analysis of the histopathological findings of the lung, cardiac and liver need core necropsies.

**Lung needle core necropsies (*N* = 62)**	***n* (%)**
Histopathological findings^*^	
Interstitial infiltrates^†^	50 (80.6)
Lymphocytes	47 (75.8)
Neutrophils	15 (24.2)
Plasma cells	11 (17.7)
Eosinophils	1 (1.6)
Histiocytes	1 (1.6)
Diffuse hyperplasia of pneumocytes	40 (64.5)
Interstitial fibrosis	38 (61.3)
Alveolar fibrosis	31 (50.0)
Alveolar infiltrates^†^	26 (41.9)
Neutrophils	15 (24.2)
Histiocytes	9 (14.5)
Lymphocytes	4 (6.5)
Plasma cells	2 (3.2)
Macrophages	2 (3.2)
Alveolar/capillary megakaryocytes	9 (14.5)
No relevant alterations (NRA)	11 (17.7)
Anatomopathological diagnoses^*^	
Diffuse alveolar damage	41 (66.1)
Exudative	12 (19.4)
Mixed	10 (16.1)
Proliferative	19 (30.6)
Acute bacterial pneumonia	12 (19.4)
Acute fibrinous and organized pneumonia	10 (16.1)
Organizing pneumonia	3 (4.8)
Necrosis	1 (1.6)
Capillaritis	1 (1.6)
No relevant alterations	11 (17.7)
**Cardiac needle core necropsies (*****N*** **=** **48)**	
***Histopathological findings*** ^*^	
Fiber hypertrophy	23 (47.9)
Cardiac fibrosis	9 (18.8)
Edema	3 (6.3)
Lymphocytic infiltrate	2 (4.2)
Mesothelial hyperplasia	1 (2.1)
Necrosis	1 (2.1)
Fat replacement	1 (2.1)
No relevant alterations	25 (52.1)
**Liver needle core necropsies (*****N*** **=** **39)**	
**Histopathological** findings^*^	
Steatosis	12 (30.8)
Cholestasis	6 (15.4)
Centrilobular necrosis	5 (12.8)
Neoplasia	3 (7.7)
Mononuclear infiltrates	2 (5.1)
Fibrosis	2 (5.1)
Cirrhosis	2 (5.1)
No relevant alterations	10 (25.6)

* *Several alterations can be detected in the same patient*.

† *Several types of inflammatory cells can be detected in the same patient*.

**Figure 5:**
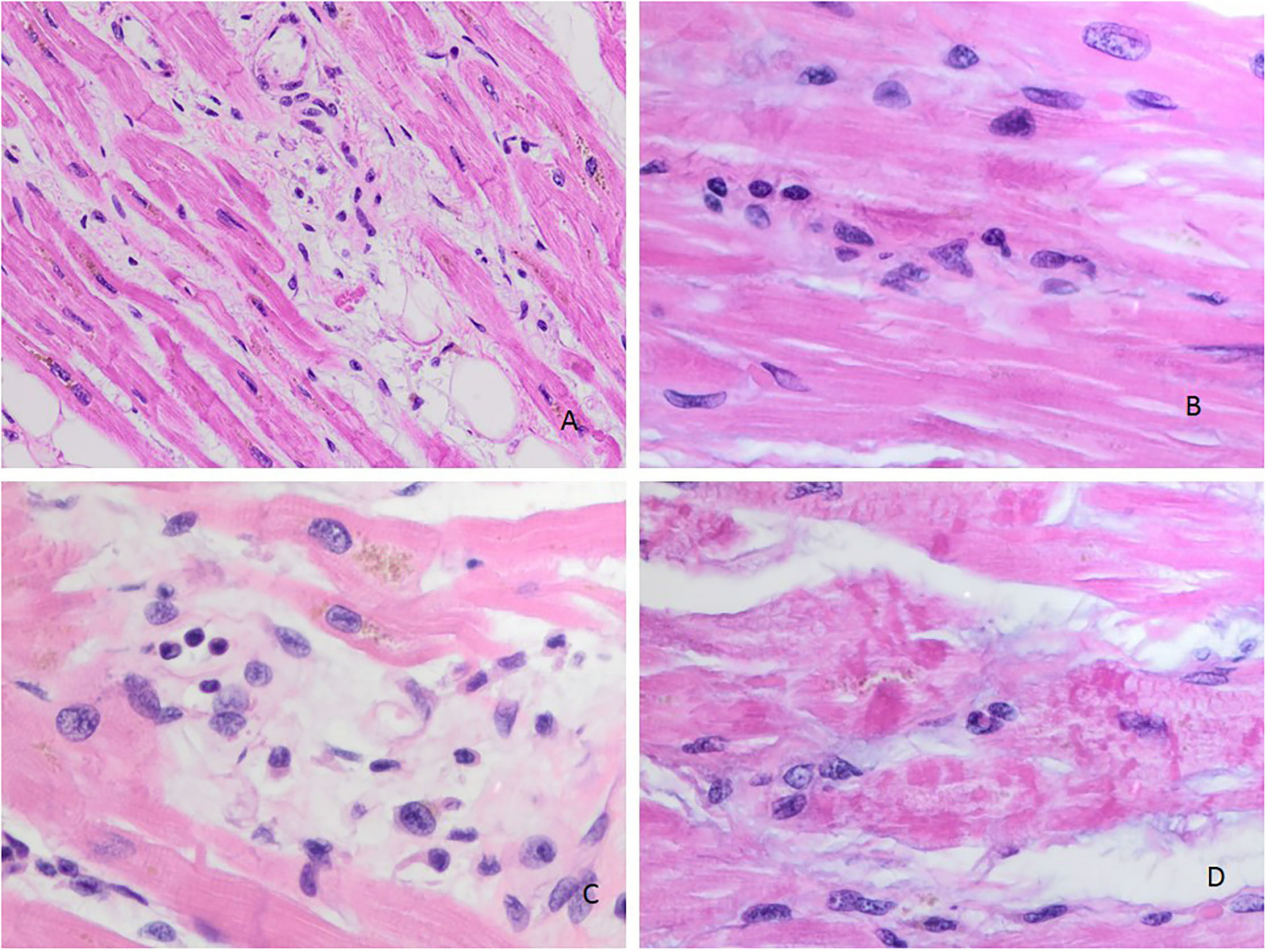
Histopathological changes in the heart. **(A)** Myocardial changes with focal edema (H&Ex200). **(B)** Mononuclear interstitial infiltrate (H&Ex400). **(C)** Destruction of myocardial cells, edema, and mononuclear cells (H&Ex400). **(D)** Necrotic myocardial cells due to ischemia (H&Ex400).

Of the 48 heart samples, 23 (47.9%) were abnormal (Figure 5). All of these showed fiber hypertrophy (47.9%), 9 (18.8%) had fibrosis, and 3, edema (Figures 5A,B). One patient presented an acute necrosis with edema and lymphocytic inflammation suggestive of myocarditis (Table 2, Figures 5C,D).

In the liver, 29 of 39 (74.4%) samples were abnormal. The main findings were steatosis (*n* = 12, 30.8%), cholestasis (*n* = 6, 15.4%) and lobular central necrosis (*n* = 5, 12.8%). Two patients had hepatic cirrhosis (Table 2, Figures 4C,D).

### SARS-CoV-2 in Pulmonary Samples

Of the 62 pulmonary postmortem samples, an RT-PCR for SARS-CoV-2 was performed in 46; 39 (84.8%) of these were positive. Six positive cases were from the 7 samples without histopathological findings (85.7%), while 33 positive cases were from 39 samples with histopathological abnormalities (84.6%). The possible association between RT-PCR positivity and the main findings in lung needle core necropsies was studied; no association was detected with any of the histopathological findings (Table 3).

**Table 3:** Analysis of RT-PCR results of in lung tissues with respect to pathological findings.

	**RT-PCR results**		***p*-Value**
**Histopathological findings**	**Positive** ** (*N* = 39) *n* (%)**	**Negative** ** (*N* = 7) *n* (%)**	
No relevant alterations	6 (15.4)	1 (14.3)	1.00
DAD	28 (71.8)	4 (57.1)	0.66
Exudative DAD	9 (23.1)	1 (14.3)	1.00
Mixed DAD	5 (12.8)	1 (14.3)	1.00
Proliferative DAD	14 (35.9)	2 (28.6)	1.00
Acute bacterial pneumonia	7 (17.9)	2 (28.6)	0.61
Acute fibrous organized pneumonia	7 (17.9)	0 (0.0)	0.57
Interstitial infiltrates	32 (82.1)	5 (71.4)	0.61
Diffuse hyperplasia of pneumocytes	28 (71.8)	3 (49.9)	0.19
Interstitial fibrosis	26 (66.7)	4 (57.1)	0.68
Alveolar fibrosis	20 (51.3)	4 (57.1)	1.00
Alveolar infiltrates	18 (46.2)	3 (49.9)	1.00

*DAD, diffuse alveolar damage*.

### Association Between Pathological Findings in Lung and Clinical, Analytical, and Radiological Findings

In the bivariable analysis, premortem ferritin values over 650 μg/l were associated with postmortem abnormalities in pulmonary samples (*n* = 7, 24.1% vs. *n* = 4, 10.0%; *p* = 0.031) (Table 4). After adjusting for age and sex, this association remained significant (AOR 4.69, 95% CI 1.05–18.11) (Table 4).

**Table 4:** Bivariable and multivariate analysis of the most relevant histopathological findings in lung needle core necropsies (*N* = 62).

	**Relevant alterations**			**Diffuse alveolar damage**		
**Bivariate analysis**	**Yes** ** (*n* = 51)** ***n* (%)**	**No** ** (*n* = 11)** ***n* (%)**	***p*-Value**	**Yes** ** (*n* = 41)** ***n* (%)**	**No** ** (*n* = 21)** ***n* (%)**	***p*-Value**
Age ≤ 80 years	28 (54.9)	4 (36.4)	0.26	25 (61.0)	7 (33.3)	**0.039**
Male sex	36 (70.6)	7 (63.6)	0.72	30 (73.2)	13 (61.9)	0.36
Obesity (BMI >30 kg/m^2^)	14 (34.1)	3 (33.3)	1.00	9 (27.3)	8 (47,1)	0.16
Hypertension	36 (70.6)	7 (6.3)	0.72	28 (68.3)	15 (71.4)	0.80
Lung disease	17 (33.3)	5 (45.5)	0.50	15 (36.6)	7 (33.3)	0.80
Charlson Comorbidity Index >3	39 (76.5)	9 (81.8)	1.00	30 (73.2)	18 (85.7)	0.35
Onset-to-death interval >15 days	29 (56.9)	5 (45.5)	0.52	24 (58.5)	10 (47.6)	0.41
Admission to ICU	18 (35.3)	2 (18.2)	0.48	17 (41.5)	3 (14.3)	**0.030**
NRA in pre-mortem chest X-ray	3 (5.9)	1 (9.1)	0.55	2 (4.9)	2 (9.5)	0.60
Interstitial infiltrate in pre-mortem chest X-ray	26 (51.0)	7 (63.6)	0.45	21 (51.2)	12 (57.1)	0.66
Pre-mortem CRP >10 mg/dl	23 (46.0)	4 (36.4)	0.74	20 (48.8)	7 (35.0)	0.31
Pre-mortem LDH >400U/l	25 (53.2)	2 (22.2)	0.15	25 (64.1)	2 (11.8)	**<0.001**
Pre-mortem ferritin >650 μg/l	36 (73.5)	4 (36.4)	**0.031**	30 (73.2)	10 (52.6)	0.12
Pre-mortem D-dimer >2.5 μg /dl	23 (52.3)	4 (50.0)	1.00	22 (56.4)	5 (38.5)	0.26
Need for NIMV	31 (60.8)	7 (63.6)	1.00	27 (65.9)	11 (52.4)	0.30
Dexamethasone	41 (80.4)	8 (72.7)	0.69	34 (82.9)	15 (71.4)	0.33
Tocilizumab	25 (49.0)	2 (18.2)	0.094	24 (58.5)	3 (14.3)	**0.001**
Death from COVID-19	45 (88.2)	9 (81.8)	0.62	37 (90.2)	17 (81.0)	0.43
**Multivariate analysis**	AOR (95% CI)^*^			AOR (95% CI)^*^		
Age ≤ 80 years	1.56 (0.37–6.66)			1.98 (0.29–13.76)		
Male sex	1.59 (0.36–6.90)			1.28 (0.19–8.55)		
Admission to ICU	–			6.88 (0.70–67.2)		
Pre-mortem LDH >400 U / l	–			**21.73 (3.22–146)**		
Pre-mortem ferritin>650 μg/l	**4.69 (1.05–18.11)**			–		
Tocilizumab	**6.91 (1.14–41.7)**			**6.91 (1.14–41.71)**		

*AOR, adjusted odds ratio; BMI, body mass index; CI, confidence interval; CRP, C-reactive protein; ICU, intensive care unit; LDH, lactate dehydrogenase; NIMV, non-invasive mechanical ventilation; NRA, no relevant alterations*.

* *The variables are adjusted for age, sex, and the significant variables from the bivariable analysis of each case. Statistically significant differences shown in bold*.

Variables associated with DAD in the bivariate analysis were age over 80 (61.0 vs. 33.3%; *p* = 0.039), ICU admission (41.5 vs. 14.3%; *p* = 0.030), premortem LDH values of 400 U/l or higher (64.1 vs. 11.8%; *p* < 0.001) and treatment with tocilizumab (58.5 vs. 14.3%; *p* = 0.001) (Table 4). In the multivariate analysis, only premortem LDH values of 400 U/l or higher (AOR 21.73; 95% CI 3.22–146) and treatment with tocilizumab (AOR 6.90; 95% CI 1.14–41.7) were associated with DAD (Table 4).

Exudative DAD was associated with male sex and high premortem LDH (Table 5). Mixed DAD was related to premortem LDH values of 400 U/l, whereas proliferative DAD was associated with age over 80 years, CCI of 3 or more, onset-to-death interval of more than 15 days, ICU admission, and treatment with tocilizumab (Table 5).

**Table 5:** Bivariate and multivariate analysis of the most relevant histopathological findings in lung needle core necropsies.

	**Exudative DAD**			**Mixed DAD**			**Proliferative DAD**		
**Univariable analysis**	**Yes** ** (*n* = 12)** ***n* (%)**	**No** ** (*n* = 50)** ***n* (%)**	***p*-Value**	**Yes** ** (*n* = 10)** ***n* (%)**	**No** ** (*n* = 52)** ***n* (%)**	***p*-Value**	**Yes** ** (*n* = 19)** ***n* (%)**	**No** ** (*n* = 43)** ***n* (%)**	***p*-Value**
Age ≤ 80 years	6 (50.0)	26 (52.0)	0.90	5 (50.0)	27 (51.9)	1.00	14 (73.3)	18 (41.9)	**0.021**
Male sex	12 (100)	31 (62.0)	0.012^*^	6 (60.0)	37 (70.2)	0.48	12 (63.2)	31 (72.1)	0.48
Obesity (BMI >30 kg/m^2^)	3 (27.3)	14 (35.9)	0.73	1 (11.1)	16 (39.0)	0.14	5 (38.5)	12 (32.4)	0.74
Hypertension	9 (75.0)	34 (68.0)	0.74	7 (30.0)	36 (69.2)	1.00	12 (63.2)	31 (72.1)	0.48
Lung disease	5 (41.7)	17 (34.0)	0.74	2 (20.0)	20 (38.5)	0.47	8 (42.1)	14 (32.6)	0.47
Charlson Comorbidity Index >3	10 (83.3)	38 (76.0)	0.72	9 (90.0)	39 (75.0)	0.43	11 (57.9)	37 (86.0)	**0.022**
Onset-to-death interval >15 days	3 (25.0)	31 (62.0)	**0.021**	4 (40.0)	30 (57.7)	0.33	17 (89.5)	17 (39.5)	**<0.001**
Admission to ICU	3 (25.0)	17 (34.0)	0.74	2 (20.0)	18 (34.6)	0.48	12 (63.2)	8 (18.6)	**0.001**
NRA in pre-mortem chest X-ray	0 (0.0)	4 (8.0)	0.58	0 (0,0)	4 (7.7)	1.00	2 (10.5)	2 (4.7)	0.58
Interstitial infiltrate in pre-mortem chest X-ray	8 (66.7)	25 (50.0)	0.30	6 (60.0)	27 (51.9)	0.74	7 (36.8)	26 (60.5)	0.086
Pre-mortem CRP >10 mg/dl	5 (41.7)	22 (44.9)	0.84	6 (60.0)	21 (41.2)	0.32	9 (47.4)	18 (42.9)	0.74
Pre-mortem LDH >400 U/l	6 (54.5)	21 (46.7)	0.64	2 (20.0)	27 (58.7)	**0.038**	11 (61.1)	16 (42.1)	0.18
Pre-mortem ferritin >650 μg/l	9 (75.0)	31 (64.6)	0.73	8 (80.0)	32 (64.0)	0.47	13 (68.4)	27 (65.9)	0.84
Pre-mortem D-dimer >2.5 μg /dl	4 (33.3)	23 (57.5)	0.14	6 (66.7)	21 (48.8)	0.47	12 (66.7)	15 (44.1)	0.12
Need for NIMV	9 (75.0)	29 (58.0)	0.34	5 (50.0)	33 (63.5)	0.49	13 (68.4)	25 (58.1)	0.44
Dexamethasone	11 (91.7)	38 (76.0)	0.43	6 (60.0)	43 (82.7)	0.20	17 (89.5)	32 (74.4)	0.31
Tocilizumab	7 (58.3)	20 (40.0)	0.25	4 (40.0)	23 (44.2)	1.00	13 (68.4)	14 (36.6)	**0.009**
Death from COVID-19	12 (100)	42 (84.0)	0.34	8 (80.0)	46 (88.5)	0.60	17 (89.5)	37 (86.0)	1.00
**Multivariate analysis**	**AOR (95% CI)** ^†^			AOR (95% CI)^*^			AOR (95% CI)^*^		
Age ≤ 80 years	1.34 (0.34–5.21)			0.91 (0.21–3.86)			1.63 (0.28–9.34)		
Male sex				0.59 (0.13–2.67)			0.57 (0.10–2.89)		
High comorbidity							2.07 (0.14–28.76)		
Onset-to-death interval >15 days	**0.19 (0.04–0.82)**						**7.85 (1.29–47.80)**		
Admission to ICU							7.05 (0.61–81.35)		
Pre-mortem LDH >400 U/l				**5.49 (1.04–29.03)**					
Tocilizumab							**7.74 (1.53–39.10)**		

*AOR, adjusted odds ratio; BMI, body mass index; CI, confidence interval; CRP, C-reactive protein; ICU, intensive care unit; BMI, body mass index; CI, confidence interval; CRP, C-reactive protein; ICU, intensive care unit; LDH, lactate dehydrogenase; NIMV, non-invasive mechanical ventilation; NRA, no relevant alterations*.

* *The variables are adjusted for age, sex, and the significant variables from the bivariable analysis of each case. The variables are adjusted for age and the significant variables in the bivariable analysis in each case. AOR: Odd ratio adjusted. Statistically significant differences shown in bold*.

In the multivariate analysis, the presence of exudative DAD was less common in patients with a long disease course (AOR 0.19; 95% CI 0.04–0.82). The presence of mixed DAD was associated with pre-mortem LDH >400 U/l (AOR 5.49, 95% CI 1.04–29.03), while the presence of proliferative DAD was associated with a long disease course (AOR 7.85, 95% CI 1.29–47.80) and treatment with tocilizumab (AOR 7.74, 95%CI 1.53–39.10; Table 5).

## Discussion

This study describes the pathological findings obtained on chest necropsy in deceased patients with COVID-19. The most common finding in the lung was DAD, especially proliferative DAD, which was most common in patients admitted to the ICU. In heart specimens, the most common finding was hypertrophy of myocardial fibers, and on liver, steatosis, and cholestasis.

With the gradual fall in conventional autopsies worldwide, the needle autopsy (also known as postmortem needle biopsy or core needle necropsy) represents a feasible alternative. As seen in our study and others in people who died from COVID-19, it can be performed at the patient's bedside, and the diagnostic accuracy is about 90% compared to conventional autopsy (16–18), making it a useful tool to better understand the cause of death in these patients.

In our study, 9 out of every 10 lung samples showed abnormal findings. The lack of abnormalities may be attributable to the non-ultrasound-guided necropsy and the low number of cores taken from each sample. In other studies, ultrasound-guided, minimally invasive autopsies with a higher number of cores (about 20–30 per organ) identified more tissue abnormalities due to SARS-CoV-2 (22).

The lung damage from COVID-19 included the presence of type II pneumocytes with nucleomegaly and prominent nucleoli, combined with an accumulation of macrophages, lymphocytes, and multinucleated giant cells, as a manifestation of DAD (9, 23, 24). In autopsies performed in patients with lung infection due to SARS in 2002 and 2003, DAD was also the main pathological finding (25, 26). Proliferative DAD was especially prevalent, representing the advanced stage of the disease. At the beginning of the disease course, SARS-CoV-2 infection causes an exudative change, transforming to proliferative DAD in some cases. The finding of exudative DAD that progresses to proliferative DAD (Figure 1C) has been seen in other studies (5, 9, 10, 21, 27, 28). In our study, DAD was present in 2 of 3 patients, consistent with previous studies; 2 of 10 had exudative DAD and mixed DAD, and 3 of 10 proliferative DAD (Figure 1C), which is consistent with other reports (5, 9, 10, 21, 27, 28).

The main histopathological finding in our study was interstitial infiltrates (8 of 10 cases), especially of lymphocytes (3 of 4 cases) (Figures 3A,B), discretely more than reported in the systematic review by Caramashi et al. (8) and other studies (10, 29, 30). Moreover, we found neutrophils and plasma cells in about 1 of 5 cases, as reported by Caramashi et al. (8) and others (10, 23).

In our study, about 2 of 3 histopathological findings showed diffuse hyperplasia of pneumocytes (Figures 1B, 3A), as also reported in the systematic reviews (5, 8) and primary studies (10, 27, 31). Moreover, interstitial and alveolar fibrosis (Figure 2C) was found in half the necropsy samples, with the fibrosing pattern arising as a consequence of DAD (8, 11, 21, 32). In other studies, fibrosis was present in most patients, and this finding was even more frequent after 3 weeks of ventilation (28). This is related to mixed and proliferative DAD, as reported in other studies. Alveolar infiltrates were seen in less than half the cases, as in other studies (8, 11, 15, 32).

In this study, acute bacterial pneumonia was present in one of five cases (Figure 2C), more than that described in Caramashi et al.'s (8) systematic review. These findings can be due to the fact that 3 of 10 deaths came after admission to the ICU with superinfection. AFOP was another relevant finding, appearing in more than 1 out of every 10 samples (Figure 4A), which is consistent with other reports (5). In COVID-19, AFOP is characterized by extensive fibrinous deposits forming balls/mounds but not hyaline membrane in their alveoli (21). In specific series of COVID-19 patients who died in the ICU, the frequency of AFOP reaches 45% of cases (28). However, in our study AFOP was not associated with ICU admission.

Organizing pneumonia secondary to a viral respiratory infection has been well-described (33), also in COVID-19 cases (30–32). These cases are probably more common than expected (34). In our research, we found histopathological findings of organizing pneumonia in 3 of 62 lung samples examined. In all cases, the interval from symptoms onset to death was more than 15 days.

Analyzing vascular injury from COVID-19, the literature describes thrombotic microangiopathy, endothelialitis and pulmonary angiogenesis. Thrombi in pre- and post-capillary vessels have been frequently described (4, 35), with thrombi usually appearing hetero-synchronously at different stages of organization (6). We found only one case of interstitial infiltrate around capillaries (capillaritis) (Figure 1A). In our study, pulmonary or alveolar hemorrhage, necrosis and vasculitis were not found, unlike other reports of autopsies in COVID-19 patients (8, 11, 12). Similarly, we did not observe Clara hyperplasia cells (36).

SARS-CoV-2 can affect cardiac tissue (8, 9, 13). In our study, half the patients contributing heart samples showed abnormalities, especially fiber hypertrophy (approx. half) and fibrosis (one in five). However, it is very difficult to establish whether the observed lesion is related to the infection or to pre-existing conditions (6, 13, 22, 37, 38) in our study nearly three-quarters of the patients were hypertensive.

One patient presented acute necrosis with edema and lymphocytic inflammation suggestive of myocarditis. This patient died from severe tachyarrhythmia due to myocarditis. Other cases of sudden myocarditis have also been reported in the literature (37, 38). Taken together, the evidence indicates that myocardial tissue is affected by SARS-CoV-2, suggesting the need for cardiological surveillance in COVID-19 survivors.

Postmortem findings from the liver have also been reported in patients who died with COVID-19 (6, 7, 9). These findings may be due to the patient's clinical status prior to infection, to liver alteration after COVID-19, or drug toxicity during SARS-CoV-2 infection management, which could increase pre-existing liver damage. For these reasons, identifying a specific histopathological pattern of liver damage in COVID-19 is challenging (9). In our study, three of every four liver samples were abnormal. Steatosis was the most common finding (30.8%), followed by cholestasis (15.4%) and lobular central necrosis (12.8%). The micro-vesicular steatosis along with mild lobular activity found in this study may be related to the viral infection, as proposed by other authors (9, 21, 39, 40). Moreover, we found several cases of a centrolobular and discrete lobular or portal inflammation, which is in line with other studies (39–41). We did not find vascular changes in the liver, as reported by other groups due to a massive lumen dilatation and partial or complete luminal thrombosis of the portal and sinusoidal vessels (41).

We did not study spleen or bone marrow tissues, although other authors have described histiocytic hyperplasia with hemophagocytosis in bone marrow needle core necropsies of people who died from severe COVID-19 (10, 41). The kidney is another organ that is severely affected in such infections, showing degenerative changes. SARS-CoV-2 may even be detected in the central nervous system (CNS), with mild neuropathological changes and pronounced inflammation in the brainstem representing the most common finding (42). However, needle core necropsies of the kidney, CNS and other organs like the testis or skin were beyond the scope of this study.

We did assess the clinical, analytical, and radiological factors related to the presence of abnormalities detected in minimally invasive autopsy. High premortem values of ferritin (650 μg/l) were associated with postmortem abnormalities in pulmonary samples. Elevated ferritin values are associated with inflammation, and higher levels of serum ferritin have been shown to be an independent predictor of in-hospital mortality (43, 44). Our results suggest that high ferritin values increase the probability of abnormalities in necropsy samples.

The finding of DAD on lung specimen was related to advanced age (>80 years), high levels of LDH (>400 UI/l), and treatment with tocilizumab. Exudative DAD and mixed DAD were, moreover, associated with LDH in the multivariable analysis, while proliferative DAD was associated with ICU admission and treatment with tocilizumab. Other authors have reported that SARS-CoV-2 virus causes acute pulmonary virus-induced senescence and subsequently fibrosis, illustrating a major mechanism of COVID-19 (45). In very old patients with immunosenescence, a SARS-CoV-2 infection may induce more alveolar senescence and subsequently DAD. Hussman (46) proposed that the inflammatory cytokines on the TNF-α/IL-6 axis and DAD (*via* cell apoptosis in respiratory epithelia and vascular endothelia) are related to elevated LDH, erythrocyte sedimentation rate (ESR), and CRP. The presence of elevated LDH represents cell necrosis activity and inflammation, which is what happens with DAD. In the literature, severe course and fatal outcomes of COVID-19 due to multi-organ injury are associated with high LDH (47). The relationship between tocilizumab and DAD may be a consequence of using tocilizumab in more severe patients rather than because tocilizumab is a risk factor for DAD in and of itself. It is known that massive pulmonary destruction is a result of highly increased levels of proinflammatory cytokines, such as tumor necrosis factor-α interleukin-6 (IL-6), IL-1β, interferon (48). Tocilizumab blocks IL-6 signaling and should reduce the pulmonary destruction and subsequent DAD (49).

The relationship between ICU admission and proliferative DAD may reside in the fact that proliferative DAD is an evolutionary stage of DAD, and this occurs in patients who have had symptoms longer and with more severity—often those admitted to the ICU, or indeed those undergoing orotracheal intubation, although this procedure was not associated with proliferative DAD. Patients with more severe disease are also the ones who have received tocilizumab, which has been associated with increased infections (50). In a case series of patients admitted to the ICU, patients who had received tocilizumab frequently presented histopathological data showing infection (50).

To summarize the correlation between the histopathology and the clinical, analytical, and radiological data and treatment, we observed that high premortem values of ferritin (650 μg/l) were associated with postmortem abnormalities in pulmonary samples. Specifically, DAD on lung specimen was related to advanced age (>80 years), high levels of LDH, and treatment with tocilizumab. Exudative DAD and mixed DAD were associated with LDH, while proliferative DAD was associated with ICU admission and treatment with tocilizumab. However, more studies are needed to corroborate this finding.

SARS-CoV-2 has been detected using different tools (immunohistochemistry for SARS-CoV-2 viral spike protein, RNA *in situ* hybridization, lung viral culture, and electron microscopy) (27). Moreover, RT-PCR analyses of histopathological specimens have been reported (31, 40, 51–54). In our study, most of the lung tissue with pathological and non-pathological findings showed direct evidence of viral RNA. The presence of the virus in lung tissue without pathological findings revealed a high viral load in these lung samples (40). The cases without viral RNA but with pathological findings may have resulted from the tests being performed a long time after the infection, with a resulting low viral RNA load that was undetectable using our procedures. These results are in accordance with other research showing that RNA is detectable in the acute phase of lung injury, but absent in the organizing phase (31). In contrast, several authors have reported the persistence of SARS-CoV-2 viral RNA in the lung even after a long postmortem interval (up to 78 days) (54).

The main strength of this study is the identification of histopathological damage caused by SARS-CoV-2 in different lung, heart, and liver tissues, by a simple postmortem needle necropsy and the clinical and analytical correlation with pathological finding. Moreover, the pathological analysis of RT-PCR SARS-CoV-2 has been scarcely reported up to now.

On the other hand, the study also has some limitations, starting with those inherent to sampling through needle core procedures and postmortem needle core necropsies (9, 55). Moreover, we did not have a control group, given the urgency of the pandemic situation, and the sample size was small. Several histopathological phenomena seen in other studies, such as pulmonary or alveolar hemorrhage, necrosis, vasculitis, arteriolar vascular microthrombi, and Clara hyperplasia cells, were either not observed or scantly observed in our samples. This could be because needle core necropsies are sometimes blind and with scanty tissue. So, the lower incidence of vascular thrombosis and endothelialitis in our series could be due to the procedures used, which are less likely to obtain tissue from pulmonary blood vessels (10). On the other hand, needle core necropsies taken in different lobes reflect well the heterogeneity of the disease and help to illustrate the variety of morphological features (10). Finally, we did not assess the expression of angiotensin-converting enzyme 2 (ACE2), which facilitates the entrance of SARS-CoV-2 in alveolar epithelial cells and capillary endothelial cells, nor did we evaluate chemokines, cytokines, or intercellular adhesion molecule 1, associated with lung damage and endotheliitis (5).

## Conclusion

There is broad consensus in the literature (8) that autopsy studies are of the utmost importance to understanding the disease features and treatment effects in COVID-19 pathophysiology. Needle autopsy has emerged as an alternative to conventional autopsy and has proven very useful to deepen our knowledge into the cause of death in these patients.

In our study, needle core necropsy shows advanced DAD as well as other findings like AFOP on lung; myocardial fiber hypertrophy and a fatal case of myocarditis on the heart; and steatosis and periportal inflammation on the liver. In the clinicopathological correlation analysis, the presence of elevated LDH values before death were associated with DAD (especially the exudative and mixed form) in the lung needle core necropsy, and admission to ICU and treatment with tocilizumab was associated with proliferative DAD in the lung needle core necropsy. Finally, the identification of SARS-CoV-2 viral load in the lung samples, with or without abnormal findings in the pathological study, was very frequent. We concur with other authors in calling for more, larger studies involving patients of different ages and physiological backgrounds.

## Data Availability Statement

The datasets analyzed during the current study are not publicly available but are available from the corresponding author on reasonable request.

## Ethics Statement

The studies involving human participants were reviewed and approved by the Ethics Committee of the Alicante General University Hospital (Spain) (PI2020-067). The patients/participants provided their written informed consent to participate in this study.

## Author Contributions

J-MR-R, CA, and IA planned and designed the project. CH-G, JP-T, F-EF-R, CM-M, PO-L, AS, AM-P, IR-M, AA-L, RG-S, LC-A, OM-P, RS-M, and EM performed the punch autopsies, acquisition, and interpretation of data. SS-O, CA, VP-C, and IA contributed on pathological analysis, acquisition, and interpretation of data. IE performed microbiological analysis, acquisition, and interpretation of data. JA-J contributed on interpretation of data radiological data. J-MR-R, CH-G, and IA write original draft. All authors reviewed the manuscript, contributed to the article, and approved the submitted version.

## Conflict of Interest

The authors declare that the research was conducted in the absence of any commercial or financial relationships that could be construed as a potential conflict of interest.

## Publisher's Note

All claims expressed in this article are solely those of the authors and do not necessarily represent those of their affiliated organizations, or those of the publisher, the editors and the reviewers. Any product that may be evaluated in this article, or claim that may be made by its manufacturer, is not guaranteed or endorsed by the publisher.

## Abbreviations

ACE2: angiotensin-converting enzyme 2
AFOP: acute fibrinous and organizing pneumonia
AOR: adjusted odds ratio
BMI: body mass index
CCI: Charlson comorbidity index
CI: confidence interval
CRP: C-reactive protein
DAD: diffuse alveolar damage
ICU: intensive care unit
LDH: lactate dehydrogenase
MERS: middle eastern respiratory syndrome
MERS-CoV: MERS-related coronavirus
NIMV: non-invasive mechanical ventilation
NRA: no relevant alterations
OR: odds ratio
RT-PCR: real-time reverse transcriptase polymerase chain reaction
SARS: severe acute respiratory syndrome
SARS-CoV-2: severe acute respiratory syndrome -coronavirus 2.
