# Supplementary material for: Pathological Findings Associated With SARS-CoV-2 on Postmortem Core Biopsies: Correlation With Clinical Presentation and Disease Course
Source: Front Med (Lausanne). 2022 Jul 7;9:874307. doi: 10.3389/fmed.2022.874307 (PMC9301383; doi:10.3389/fmed.2022.874307)
Supplement: Supplementary file 1 [file Table_1.docx]

**Table 1S**. Descriptive analysis of the population.

| **Variables** | **Patients (N = 71)** |
| --- | --- |
| **Demographic variables** |  |
| Age in years, median [IQR]  > 80 years, n (%)  Men, n (%)  Race/ethnicity  White, n (%)  Latin American, n (%)  North African, n (%)  Institutionalized in residence, n (%)  COVID-19 acquired during admission, n (%) | 81 [69;87]  36 (50.7)  48 (67.6) |
|  | 65 (91.5)  5 (7.0)  1 (1.4) |
|  | 7 (9.9) |
|  | 11 (15.5) |
| **Comorbidities** |  |
| Body mass index^a^ in kg/m^2^, mean ± SD  Overweight (BMI> 25 kg/m^2^), n (%)  Obesity (BMI> 30 kg/m^2^), n (%)  Smoker or ex-smoker^b^, n (%)  > 40 pack-years, n (%)  Arterial hypertension, n (%)  Diabetes mellitus, n (%)  Pulmonary disease, n (%)  Cardiovascular disease, n (%)  Immunosuppression, n (%)  Age-adjusted Charlson comorbidity index (CCI), median [IQR]  High comorbidity (> 3 CCI), n (%)  Clinical Frailty Scale (CFS)^c^, median [IQR]  Frail (≥4 CFS), n (%) | 28.82 ± 4.43  47 (66.2)  20 (28.2)  24 (34.7)  9 (12.7) |
|  | 51 (71.8)  31 (43.7)  24 (33.8)  28 (39.4)  6 (8.5)  6 [4;8]  57 (80.3)  4 [3;6]  47 (67.1) |
| **Administrative variables** |  |
| Interval from symptoms onset to admission, days, median [IQR]  Interval from symptoms onset to death, days, median [IQR]  Onset-to-death interval > 15 days, n (%)  Length of hospital stay, median [IQR]  Admission to ICU, n (%)  Length of ICU stay, mean ± SD  Need for orotracheal intubation  Days of orotracheal intubation, mean ± SD | 5 [2;7]  17 [8;27]  39 (54.9) |
|  | 11 [5;20]  21 (29.6)  20 ± 13  20 (28.2)  20 ± 13 |
| **Clinical presentation at admission, n (%)** |  |
| Fever  Dry cough  Productive cough  Dyspnea  Asthenia  Myalgia  Diarrhea  Confusional syndrome  Anosmia or ageusia | 32 (45.1)  35 (49.3)  12 (16.9)  51 (71.8)  22 (31.0)  5 (7.0)  5 (7.0)  13 (18.3)  3 (4.2) |
| **Vital signs on admission** |  |
| Temperature, °C, mean ± SD  Temperature > 38°C, n (%)  Oxygen saturation^d^, % O_2_ (O_2_ sat), median [IQR]  O_2_ sat <90%, n (%)  Respiratory rate^e^, bpm, median [IQR]  Tachypnea (> 20 bpm), n (%)  Systolic blood pressure (SBP)^f^ in mmHg, mean ± SD  Hypotension (SBP <100 mmHg), n (%)  Diastolic blood pressure (DBP)^g^ in mmHg, median [IQR]  Heart rate (HR) in bpm, mean ± SD  Tachycardia (> 100 bpm), n (%)  PaO_2_/FiO_2_^h^ ratio, mean ± SD  PaO_2_/FiO_2_ <300, n (%) | 36.8 ± 1.1  12 (16.9)  91.5 [84;94]  27 (40.9)  22 [20;31.5]  29 (60.4)  130 ± 26  6 (8.6)  71 [63;84]  92 ± 24  24 (33.8)  289 ± 109  34 (50.7) |
| **Pre-mortem chest X-ray findings, n (%)*** |  |
| No acute pathological alterations  Unilateral or bilateral interstitial infiltrates  Bilateral pneumonia  Lobar pneumonia  Acute lung edema | 8 (11.3)  36 (50.7)  25 (35.2) |
|  | 4 (5.6)  6 (8.5) |
| **Treatment, n (%)** |  |
| Need for non-invasive mechanical ventilation†  High-flow nasal cannula oxygen  Continuous positive airway pressure  Bilevel positive airway pressure  Antibiotic therapy (> 48 h)  Corticotherapy  Dexamethasone  Methylprednisolone  Tocilizumab  Remdesivir  Convalescent plasma | 44 (62.0) |
|  | 34 (47.9)  11 (15.5)  1 (1.4)  63 (88.7)  62 (87.3)  55 (77.5)  7 (9.8)  30 (42.3)  5 (7.0)  5 (7.0) |
| **Cause of death, n (%)** |  |
| Secondary to COVID-19  Cardiovascular  Other infections  Other causes | 63 (88.7)  2 (2.8)  4 (5.6)  2 (2.8) |

IQR: interquartile range. SD: standard deviation. ICU: intensive care unit. IOT: orotracheal intubation. PaO_2_: arterial oxygen partial pressure in mmHg. FiO_2_: fractional inspired oxygen. NIMV: non-invasive mechanical ventilation. * Some patients had more than one radiological pattern. †Some patients received more than one type of NIMV at different time of disease. Missing: ^a^16, ^b^1, ^c^1, ^d^5, ^e^23, ^f^1, ^g^1, ^h^4.

**Table 2S**. Descriptive analysis of the laboratory findings

|  | **At admission** | **Before death** |
| --- | --- | --- |
| Glomerular filtration rate (GFR) by CKD-EPI, median [IQR]  GFR <60 by CKD-EPI, n (%)  Urea in mg/dl, median [IQR]  Potassium (K) in nmol/l, median [IQR]  K> 4.5, n (%)  C-reactive protein (CRP) in mg/dl, median [IQR]  CRP> 10, n (%)  Pro-BNPj in pg/ml, median [IQR]  Troponin T in ng/dl, median [IQR]  Procalcitonin in ng/dl, median [IQR]  Glutamic-oxaloacetic transaminase in U/l, median [IQR]  Glutamic-pyruvic transaminase in U/l, median [IQR]  Creatinine kinase in U/l, median [IQR]  Lactate dehydrogenase (LDH) in U/l, median [IQR]  LDH> 400, n (%)  Ferritin in µg/l, median [IQR]  Ferritin> 650, n (%)  Leukocytosis by µl, median [IQR]  Lymphocytes by µl, median [IQR]  Lymphopenia (lymphocytes <1000), n (%)  D-dimer in µg/dl, median [IQR]  D-dimer> 2.5, n (%)  Interleukin 6 (IL-6) in pg/ml, median [IQR] | 48.53 [25.61;79.13]  32 (45.1) |  |
|  | 71 [50;107]  4.3 [3.9;4.9]  28 (39.4)  10.22 [5.28; 22.25]^a^  37 (52.9)  640 [406; 7431]^b^  39 [19;60]^c^  0.28 [0.13; 0.82]^d^  40 [27;56]^e^  20 [15; 41.5]^f^  78 [53; 199]^g^  336 [260;500]^h^  25 (37.3)  1061 [381; 2136]^i^  45 (64.3)  7285 [4847; 10697]^j^  670 [450;1110]  50 (70.4)  1.43 [0.87; 4.32]^k^  21 (31.8)  88 [51; 180]^l^ | 4.5 ± 0.7^m^  36 (50.7)  8.74 [3.08; 19.57]^n^  31 (44.3)  3770 [901; 10123]^o^  54 [30;88]^p^  36 [27; 58]^q^  31 [20; 58]^r^  63 [31; 130]^s^  404 [325; 572]^t^  31 (50.0)  1596 [513; 2524]^u^  46 (68.7)  2.65 [1.24; 5.08]^v^  31 (51.7) |

IQR: interquartile range. SD: standard deviation. Missing: ^a^1, ^b^8, ^c^9, ^d^3, ^e^2, ^f^1, ^g^7, ^h^4, ^i^1 ,^j^1, ^k^5, ^l^30, ^m^1, ^n^1, ^o^12, ^p^19, ^q^1, ^r^1, ^s^2, ^t^9, ^u^4, ^v^10.
